# Supplementary material for: Immunomodulatory Potential of a Composite Amniotic Membrane Hydrogel for Wound Healing: Effects on Macrophage Cytokine Secretion
Source: Biomedicines. 2025 Oct 21;13(10):2574. doi: 10.3390/biomedicines13102574 (PMC12562051; doi:10.3390/biomedicines13102574)
Supplement: Supplementary file 1 [file biomedicines-13-02574-s001.zip › biomedicines-3851884-supplementary.pdf]

Determination of Encapsulation Efficiency and Drug Loading Capacity of DAPT/VEGF Microspheres

Approximately 100 mg of DAPT or VEGF microspheres were accurately weighed and placed in a conical flask. Exactly 50 mL of anhydrous ethanol was added precisely, and the flask was weighed. The mixture was sonicated for 30 minutes. After sonication, anhydrous ethanol was added to compensate for any weight loss. The solution was then filtered through a 0.45 μm microporous membrane, and the subsequent filtrate was collected for the quantification of DAPT or VEGF content. The encapsulation efficiency (EE) and drug loading (DL) capacity were calculated using the following formulas:

Drug Loading (DL) = (Amount of drug in microspheres / Weight of microspheres) × 100%

Encapsulation Efficiency (EE) = (Amount of drug in microspheres / Total amount of drug used in formulation) × 100%

The results showed that the encapsulation efficiencies for DAPT and VEGF microspheres were 79.42% and 83.24%, respectively, and the drug loading capacities were 45.04% and 48.32%, respectively.

In Vitro Release Study of DAPT/VEGF Microspheres

An in vitro release study was conducted using phosphate-buffered saline (PBS, pH 7.4) as the release medium in a constant-temperature water bath shaker at 37 °C and a constant speed of 100 rpm. Approximately 135 mg of DAPT or VEGF microspheres (n=6 for each) were accurately weighed, placed into pre-treated dialysis bags, and dispersed in 5 mL of release medium. Both ends of the dialysis bags were tightly sealed and attached to the stirring paddles of the dissolution apparatus. Each dissolution vessel contained 95 mL of release medium. Samples (1 mL) of the release medium were withdrawn at predetermined time intervals (0.25, 0.75, 1, 2, 4, 6, 10, 16, and 24 hours). An equal volume (1 mL) of fresh, pre-warmed blank medium was immediately added to the vessel to maintain a constant volume. The withdrawn samples were filtered through a 0.45 μm organic filter membrane. The DAPT content at each time point was determined using High-Performance Liquid Chromatography (HPLC), while the VEGF content was measured using an Enzyme-Linked Immunosorbent Assay (ELISA).

The in vitro release results indicated that the cumulative release rates of DAPT and VEGF from the microspheres over 24 hours were 95.3% and 90.59%, respectively, demonstrating a certain sustained-release effect.
